# Expression profiling identifies key genes and biological functions associated with eosinophilic esophagitis in human patients

**DOI:** 10.3389/falgy.2023.1239273

**Published:** 2023-08-24

**Authors:** Holly A. Morrison, Kacie J. Hoyt, Christina Mounzer, Hannah M. Ivester, Barrett H. Barnes, Bryan Sauer, Emily C. McGowan, Irving C. Allen

**Affiliations:** ^1^ Department of Biomedical Sciences and Pathobiology, Virginia-Maryland College of Veterinary Medicine, Virginia Tech, Blacksburg, VA, United States; ^2^ Graduate Program in Translational Biology, Medicine, and Health, Virginia Polytechnic Institute and State University, Roanoke, VA, United States; ^3^ Division of Pediatric Gastroenterology/Nutrition, Department of Pediatrics, University of Virginia School of Medicine, Charlottesville, VA, United States; ^4^ Division of Gastroenterology and Hepatology, Department of Medicine, University of Virginia School of Medicine, Charlottesville, VA, United States; ^5^ Division of Allergy and Clinical Immunology, Department of Medicine, University of Virginia School of Medicine, Charlottesville, VA, United States; ^6^ Division of Allergy and Clinical Immunology, Johns Hopkins University School of Medicine, Baltimore, MD, United States; ^7^ Department of Basic Science Education, Virginia Tech Carilion School of Medicine, Roanoke, VA, United States

**Keywords:** human esophageal biopsy, mucin, keratinization, epidermal differentiation complex, epithelial barrier

## Abstract

**Introduction:**

Eosinophilic Esophagitis (EoE) is a chronic allergic disease characterized by progressive inflammation of the esophageal mucosa. This chronic inflammatory disorder affects up to 50 per 100,000 individuals in the United States and Europe yet is limited in treatment options. While the transcriptome of EoE has been reported, few studies have examined the genetics among a cohort including both adult and pediatric EoE populations. To identify potentially overlooked biomarkers in EoE esophageal biopsies that may be promising targets for diagnostic and therapeutic development.

**Methods:**

We used microarray analysis to interrogate gene expression using esophageal biopsies from EoE and Control subjects with a wide age distribution. Analysis of differential gene expression (DEGs) and prediction of impaired pathways was compared using conventional transcriptome analysis (TAC) and artificial intelligence-based (ADVAITA) programs. Principal Components Analysis revealed samples cluster by disease status (EoE and Control) irrespective of clinical features like sex, age, and disease severity.

**Results:**

Global transcriptomic analysis revealed differential expression of several genes previously reported in EoE (*CCL26, CPA3, POSTN, CTSC, ANO1, CRISP3, SPINK7*). In addition, we identified differential expression of several genes from the *MUC* and *SPRR* families, which have been limited in previous reports.

**Discussion:**

Our findings suggest that there is epithelial dysregulation demonstrated by DEGs that may contribute to impaired barrier integrity and loss of epidermal cell differentiation in EoE patients. These findings present two new gene families, *SPRR* and *MUC*, that are differentially expressed in both adult and pediatric EoE patients, which presents an opportunity for a future therapeutic target that would be useful in a large demographic of patients.

## Introduction

Eosinophilic Esophagitis (EoE) is a chronic allergic disease that is characterized by progressive inflammation of the esophageal mucosa, which in severe cases may lead to food impaction and esophageal stricture (1, 2). This eosinophil-associated gastrointestinal disorder (EGID) affects up to 50 per 100,000 individuals in the United States and Europe and can lead to significant impairment in quality of life if untreated, demonstrating the need for effective and targeted treatments (3). Epidemiologic data suggests that the incidence of EoE is exponentially increasing, with some studies reporting up to a 100-fold increase in recent years (4). Risk factors ranging from geographical location and heritable traits to early antibiotic exposure have been implicated in the pathogenesis of EoE (5). EoE affects both adult and pediatric populations, although several differences between the character of the disease in these groups have been reported (6, 7). Children tend to display more acute manifestations of inflammation-like exudates and furrows, whereas adults tend to have more fibrostenotic signs of disease, such as strictures (8). Identifying biologic factors that are shared in both the pediatric and adult EoE populations would pave the way for novel diagnostic and treatment targets for the wide spectrum of this disease.

EoE is diagnosed by presence of symptoms of esophageal dysfunction (dysphagia, food impaction, heartburn, chest pain), histologic confirmation by H&E stain of ≥15 eosinophils per high power field (eos/hpf), and the exclusion of other causes of esophageal eosinophilia (4).The treatment goal is symptomatic management and prevention of esophageal remodeling. First-line treatment for EoE is pharmacologic intervention proton pump inhibitors/corticosteroids or empiric elimination diets (9). Recently, several clinical trials repurposing biologics used for other indications in the treatment of EoE have been evaluated with variable success (10). Dupilumab (IL-4R*α* antagonist) completed its Phase III trial in May 2022 and is currently FDA approved (11). Although originally promising, Mepolizumab (IL-5 antibody) did not meet its primary endpoint in a Phase III study for EoE (12). Monoclonal antibodies targeting IL-13, anti-TNF, and anti-TSLP are still ongoing (10). Limited success therefore offers an opportunity for identifying new EoE-specific markers for potential targeted therapies specific to a disease process.

Seminal work by Rothenberg described the “EoE transcriptome”, including 574 transcripts associated with pathogenesis, including overexpression of the eosinophil-specific chemoattractant eotaxin-3 (*CCL26*) (13). Subsequent work delineated the molecular pattern of epithelial barrier dysfunction in EoE with downregulated filaggrin (*FLG*) and desmoglein (*DSG1*) (14, 15). The *SPINK* family is also dysregulated in EoE patients, likely contributing to the permeability of the epithelium (16). Disruption of the epithelial barrier promotes the T_H_2response in the esophagus by directly releasing TSLP, IL-33, and IL-25, and enabling relevant antigens to penetrate the esophageal mucosa.

Consistently described as an allergic disease, the presence of T_H_2 cells and cytokines have been identified in EoE patients. T_H_2 associated cytokines IL-4, IL-5, and IL-13, were induced in patients with active EoE by milk, supporting the allergic etiology of EoE (17). This is corroborated by genetic associations that describe *TSLP* as a promoter of the T_H_2 response in EoE subjects (14). This overactive T_H_2 response, particularly IL-13 production, further induces an epithelial response and diminishes barrier function as EoE patients have overall decreased *DSG1* and decreased expression of genes related to epithelial structural genes, including *FLG*, *IVL,* and the SPRR gene family (18). These genes also comprise the epidermal differentiation complex (EDC) and include the expression of the cytokeratins *KRT4* and *KRT13* in the suprabasal zone, which contain more differentiated cells. Likewise, E-cadherin is expressed in both the basal and suprabasal layers by epithelial cells (18). Therefore, the transcriptome of EoE patients is characterized by a unique esophageal gene expression signature that is indicative of altered esophageal tissue differentiation, impaired barrier function, and overzealous allergic responses.

Regulators of local epithelial barrier dysfunction and the T_H_2 response have been well described by molecular studies. In this manuscript, we confirm commonly identified transcriptomic patterns in the pathogenesis of EoE. Further, we extend these results by highlighting the *MUC* and *SPRR* families, which were highly represented among the most differentially expressed gene families in our dataset. These findings may underscore pathways that have yet to be investigated in the development of EoE and represent a potential target for future therapeutic studies.

## Materials & methods

### Subject recruitment

A total of 24 (12 EoE, 12 Control) subjects were enrolled into this study with informed consent from all participants and/or guardians. All studies were conducted under the approval of the Institutional Review Board. EoE subjects were individuals with suspected EoE scheduled for esophagogastroduodenoscopy (EGD) for diagnostic confirmation. Control subjects were individuals undergoing EGD for investigative purposes or other non-inflammatory etiologies. All patients selected were undergoing an initial EGD and not yet prescribed therapeutics for EoE. All relevant data, including health information, laboratory results, pathology results, endoscopy scores, treatment, and questionnaire responses were recorded.

### Sample collection

Tissue samples were obtained during clinically indicated EGD procedures with clinical assessment of Eosinophilic Esophagitis Endoscopic Reference Score (EREFS). Biopsies collected from endoscopically visible areas of active inflammation and healthy mucosa in subjects and controls, respectively. Definitive diagnosis of EoE was made using clinical, endoscopic, and histologic metrics per current guidelines (4), including symptoms of esophageal dysfunction, presence of ≥15 eos/hpf, and exclusion of other potential causes of esophageal eosinophilia. Controls were defined as patients who underwent an EGD for symptoms suggestive of EoE but not confirmed with endoscopic or histologic assessment. Samples were then stored in RNAlater (Qiagen) until further processing.

### Variable definitions

Active disease was defined as active inflammation based on endoscopy. Disease duration was categorized by years since diagnosis in 3 groups: <1, 1–5, >5 years. Eosinophilic Esophagitis Reference Score (EREFS) disease activity summary score was recorded at the time of biopsy (19). Eosinophilia grading was developed using eos/hpf histologic data: mild = 0–6 eos/hpf, moderate = 7–15 eos/hpf, severe = 15–30 eos/hpf, and profound = 31 + eos/hpf.

### Tissue processing and microarray analysis

Biopsy samples were homogenized in RLT buffer (Qiagen). DNA, RNA, and protein were extracted using the AllPrep kit per manufacturer's protocol (Qiagen). RNA was assessed for quality using NanoDrop™ and stored at −80°C. 10 µl RNA (50 ng/µl) were plated on a barcoded 96-well plate provided by Thermo Fisher Scientific. Microarray analysis using Clariom™ S Assay for human samples was performed by Thermo Fisher Scientific. Plate normalization, hybridization, and processing are performed as part of the throughput at Thermo Fisher Scientific. Finalized data was distributed in the format of CEL files.

### Data analysis

Population descriptors were expressed as frequency (number and percentage) and median ± IQR, as appropriate. Microarray analyses were performed using the Thermo Fisher Transcriptome Analysis Console (TAC). CEL files were imported to the TAC software and initial quality control evaluation was conducted. Samples passed quality control for three parameters, including labeling controls, hybridization controls, and positive vs. negative AUC thresholds. Principal Components Analysis (PCA) were used to determine if samples clustered based on disease status or other clinical metrics. Differential gene expression (DEG) was used to determine gene families and clusters that were differentially up-/down-regulated between EoE and Control groups, with significance defined as ≥2, ≤−2-fold change.

Analysis using ADVAITA Bioinformatics iPathwayGuide, which is an AI-based platform for predictive pathway analysis (ADVAITA Bioinformatics, Ann Arbor, MI, USA), was conducted in parallel. All significant DEGs were used as input to determine the significantly impacted pathways with pathway annotations being derived from the Kyoto Encyclopedia of Genes and Genomes (KEGG) database. Significant pathways (false discovery rate adjusted *p* values <0.1) were presented as –log (*p*-value). The top significantly enriched Gene Ontology (GO) biological processes and cellular components are reported as -log10 (*p*-value). Significant DEGs for the SPRR and MUC family were input into g:Profiler (https://biit.cs.ut.ee/gprofiler/gost) for functional profiling with statistically significant related Reactome pathways reported as -log10 (*p*-value).

## Results

### Patient characteristics

This study utilized tissue samples from 12 EoE and 12 Control subjects. The EoE group was 33.3% female compared to 83.3% female in the Control group (Table 1). The age distribution was comparable with a median age of 18 years in both EoE (range: 5–31 years) and Control (range: 5–49) groups. The study population overall was predominantly white (83.3%), non-Hispanic Latino (83.3%). None of the Control samples had evidence of disease activity on biopsy, whereas all the EoE subjects had evidence of active disease at the time of sampling. Half of the EoE subjects had a disease duration of greater than 5 years, 33.3% 1–5 years, and 16.7% less than 1 year. The median disease severity based on endoscopic reference score assessing EREFS in the EoE group was 3.5 (range: 1–6). Histological grading of the EoE biopsy samples revealed a median eosinophilia count of 31 eos/hpf (range: 15–92). The transcriptomic data was analyzed for the impact of these clinical features on the clustering of samples using principal component analysis (PCA). The samples strongly clustered by disease category, moderately clustered by disease duration, moderately clustered based on sex, and no defined clustering based on age category (Supplementary Figures S1, S2). Therefore, disease status better defines the clustering observed, with their being stratification given disease duration. Clustering based by sex may likely be explained by EoE being nearly 4 times more prevalent in males than females (20). We anticipate that clustering effects would become stronger given a larger sample population. Based on the appearance of these clusters, we further explored sample transcriptomics to better understand which gene families are driving the clusters.

**Table 1:** Demographic and clinical characteristics of subjects.

Characteristic	EoE (*n* = 12)	Control (*n *= 12)
Sex- No. (%)		
Female	4 (33.3)	10 (83.3)
Male	8 (66.7)	2 (16.7)
Age- median (range)	18 (5–31)	18 (5–49)
Age category- No. (%)		
0–10 year	1 (8.3)	1 (8.3)
11–20 year	6 (50.0)	6 (50.0)
21–30 year	3 (25.0)	1 (8.3)
31–40 year	2 (16.7)	3 (25.0)
41–50 year	–	1 (8.3)
Race- No. (%)		
White	9 (75.0)	11 (91.7)
Bi/multi racial	1 (8.3)	–
African American	–	1 (8.3)
American Indian	–	–
Asian	–	–
Native Hawaiian	–	–
Not identified	2 (16.7)	–
Ethnicity- No. (%)		
Hispanic/latino	2 (16.7)	1 (8.3)
Non-hispanic latino	9 (75.0)	11 (91.7)
Unknown	1 (8.3)	–
Active disease- No. (%)	12 (100)	0 (0)
Disease duration- No (%)		
<1 year	2 (16.7)	7 (58.3)
1–5 year	4 (33.3)	2 (16.7)
>5 year	6 (50.0)	3 (25.0)
EREFS- median (range)	3.5 (1–6)	0 (0–2)
Eos/hpf- median (range)	31 (15–92)	0 (0–2)

EoE, eosinophilic esophagitis; Eos/hpf, eosinophils per high power field; EREFS, endoscopic reference score assessing edema, rings, exudates, furrows, and strictures.

### Global transcriptomic analysis shows sample clustering by disease state

**Figure 1:**
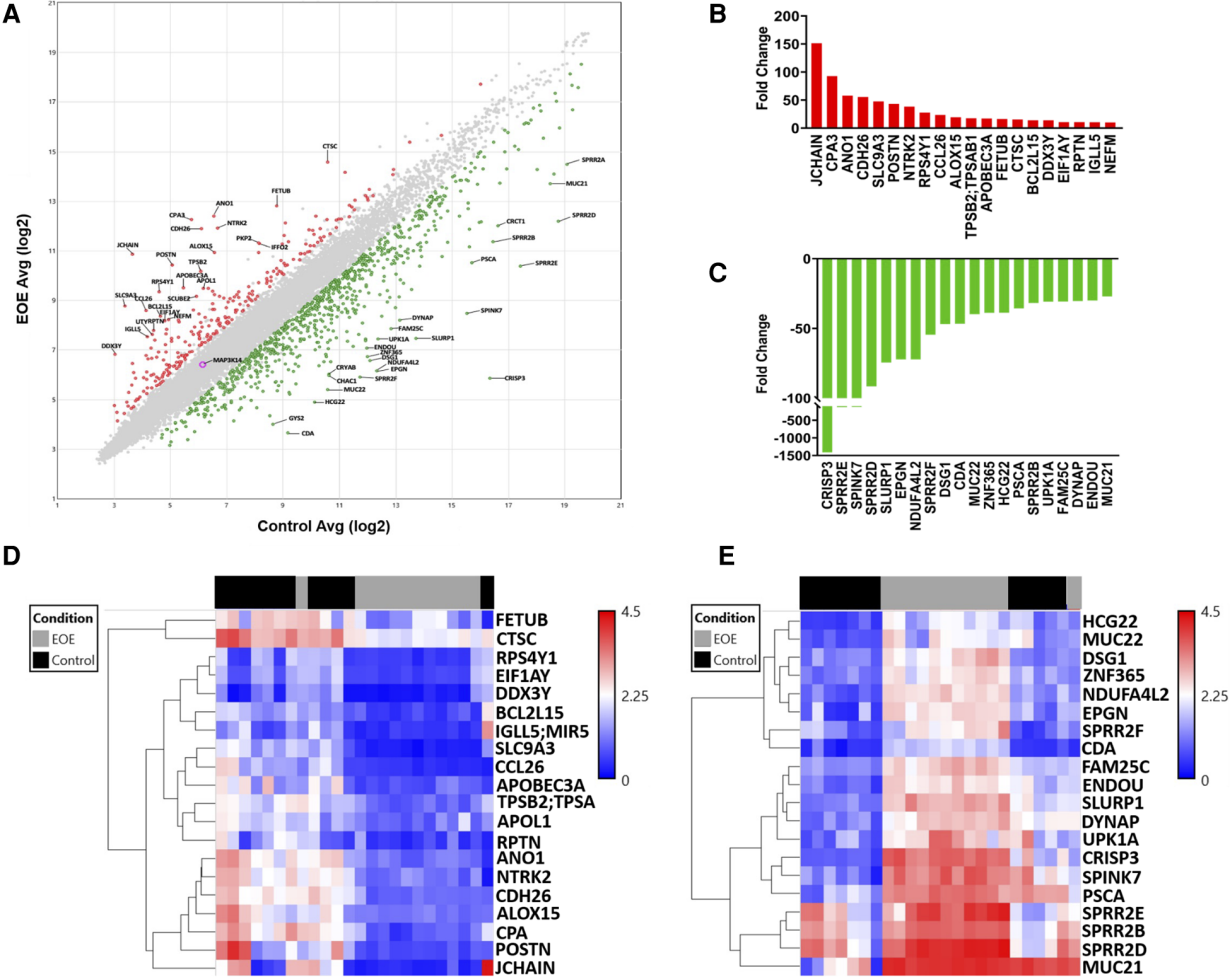
Global transcriptomic analysis of esophageal biopsies. (**A**) Scatter plot of differentially expressed genes between EoE and control subjects. (**B**) Top 20 upregulated genes. (**C**) Top 20 downregulated genes. (**D**) Heatmap depicting hierarchical clustering of top 20 upregulated genes. (**E**) Heatmap depicting hierarchical clustering of top 20 downregulated genes.

Microarray analysis returned data on 21,448 genes, 1,059 of which were DEGs between EoE and Control samples. Scatterplot analysis of the DEGs revealed 806 (76.1%) were up-regulated and 253 (23.9%) were down-regulated (Figure 1A). The top 20 up-regulated (Figure 1B) and down-regulated genes (Figure 1C) represented a diverse repertoire of functions. The most differentially expressed up-regulated genes were *JCHAIN* (151.4-fold), *CPA3* (92.8-fold), *ANO1* (57.9-fold), and *CDH26* (55.7-fold) which all had a greater than 50-fold change increase compared to Controls. Overall, the down-regulated genes were more differentially expressed with 3 genes having more than a 100-fold decrease compared to Controls: *CRISP3* (−1411.9-fold), *SPRR2E* (−127.7-fold), and *SPINK7* (−123.9-fold). Hierarchical clustering of up-regulated genes displayed close relationships between all but one of the Control samples, suggesting a similar transcriptomic profile. Similarly, one EoE sample clustered more tightly with the Control group than the EoE group (Figure 1D). Interestingly, hierarchical clustering of the down-regulated genes displayed 2 distinct subgroups of EoE patients, while the Control samples remained tightly clustered (Figure 1E). The Control sample that clustered with the EoE group in the up-regulated gene heatmap remained clustered with EoE samples in the down-regulated heatmap.

### Transcriptomic data aligns with commonly reported EoE genes and pathways

**Figure 2:**
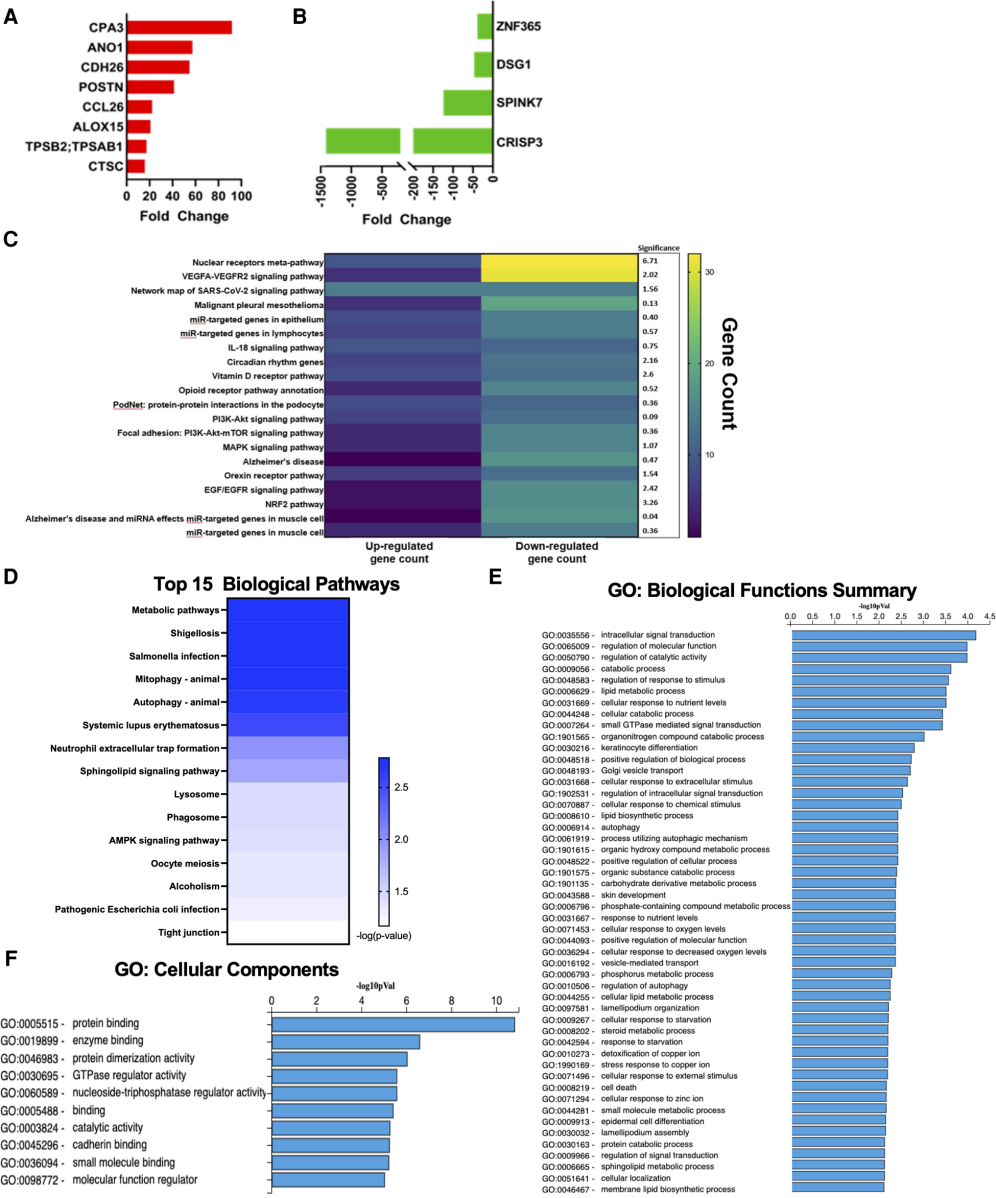
Genes commonly associated with EoE. (**A**) Upregulated genes commonly associated with EoE. (**B**) Downregulated genes commonly associated with EoE. (**C**) Heatmap of top pathways underlying transcriptomic expression indicated by gene count. (**D**) Top 15 biological pathways. (**E**) GO: biological functions summary. (**F**) GO: cellular components.

Our dataset confirmed several classic genetic markers of EoE. Among the top 20 up-regulated genes, 8 have been previously associated with EoE. Notably eotaxin-3 (*CCL26*), an eosinophil chemotactic chemokine, which has been extensively reported as a strong driver of EoE (13, 21) was represented in this group (Figure 2A). These up-regulated genes have also been reported to play a breadth of roles in EoE pathophysiology including: mast cells (*CPA3*), tissue remodeling (*POSTN*; *CTSC*), and ion channel function/inflammation (*ANO1*; *ALOX15*) (22, 23) (Figure 2A). The most highly down-regulated gene (*CRISP3*) has been described as impacting epithelial function in EoE patients (16). *SPINK7*, down-regulated by 132-fold in our dataset, has been implicated in regulating esophageal barrier maintenance and down-regulation may be associated with EoE pathogenesis (16). The downregulated genes have also been associated with cell adhesion and inflammation in EoE, such as *DSG1* (14, 15) (Figure 2B). Several of the top pathways based on gene count were associated with barrier cell function and integrity (VEGFA-VEGFR2; Endothelin; EGF/EGFR) (Figure 2C). Tight junctions are a critical signaling component of barrier function and were also implicated in pathway analysis performed by ADVAITA iPathwayGuide (Figure 2D). The IL-18 signaling pathway, which has been linked to mast cell recruitment in food allergen induced EoE, was also represented in the pathway analysis (24) (Figure 2C). Autophagy was one of the top pathways to be implicated by the dysregulated transcriptome (Figure 2D). This is consistent with a previous study that found autophagy to be a potential drug target and critical for maintaining metabolic homeostasis within the esophageal epithelia following exposure to pro-inflammatory cytokines (25). Of interest, metabolic pathways was the top pathway predicted with a -log10 *p*-value of 2.78 (Figure 2D). Dysregulation of these predicted pathways is expected to impair functions largely related to intercellular signal transduction, catalytic activity, lipid metabolism, response to stimuli, and keratinocyte differentiation (Figure 2E). Pathologic activity is expected to interfere with protein binding, enzyme binding, and cadherin binding (Figure 2F). Defects in cadherin binding is another mechanism that may impair barrier function in EoE patients. A study conducted by Doshi et al. found that pediatric EoE patients had decreased membrane bound E-cadherin (26). Therefore, loss of E-cadherin is predicted to decrease barrier integrity by interfering with tight junction interactions. Our findings recapitulate the well-described etiology of EoE as a food allergen directed destruction of the esophageal barrier.

### Altered gene expression in genes related to keratinization

**Figure 3:**
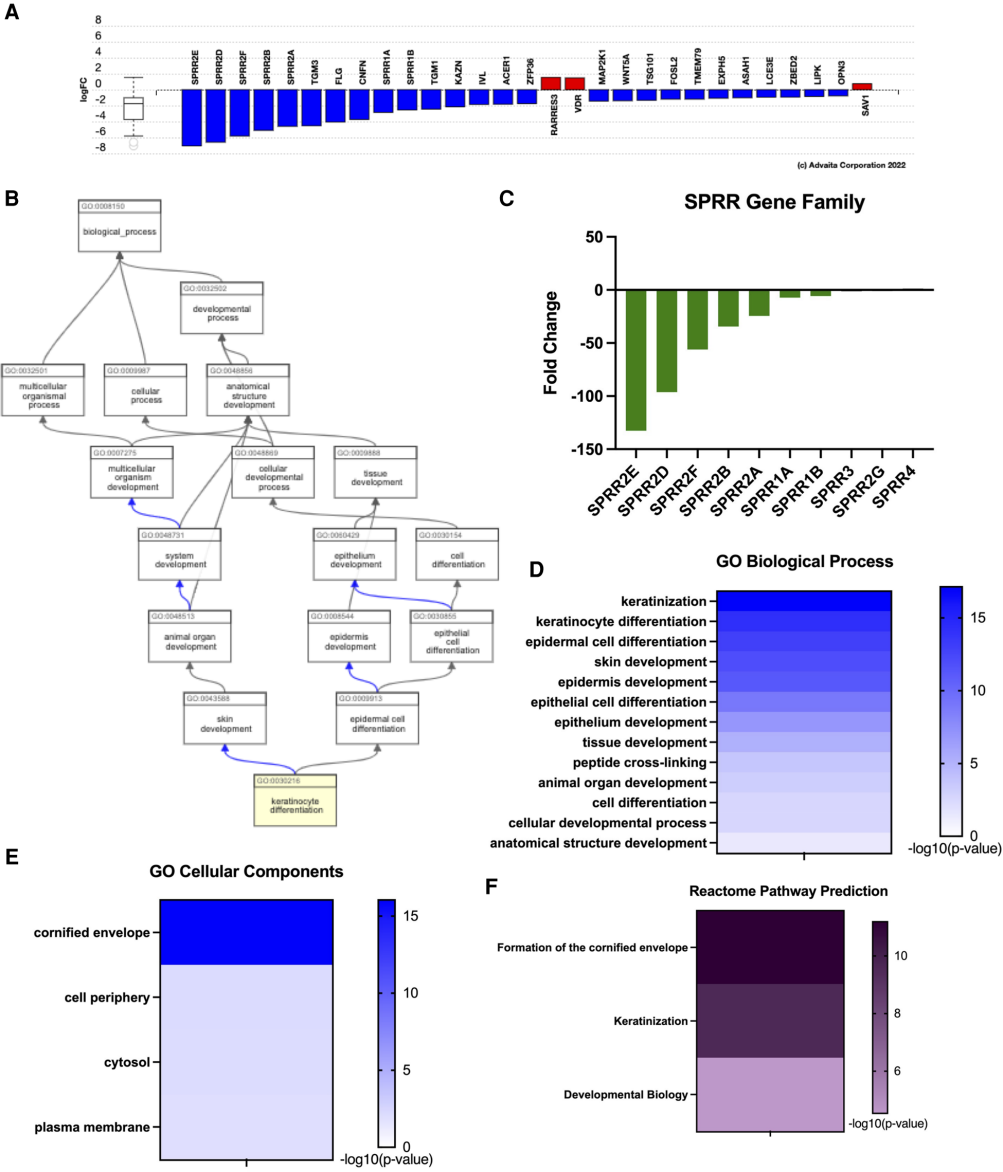
Eoe characterized by impaired keratinization given downregulated SPRR genes. (**A**) Dysregulation of genes related to keratinization. (**B**) Downstream effects of keratinocyte differentiation. (**C**) SPRR gene expression, (**D**) GO: biological processes, (**E**) GO: cellular components, (**F**) reactome pathway prediction.

Previous work assessing esophagus-specific transcripts following whole exome sequencing described impaired regulation of epithelial differentiation and keratinization (27). Consistently, keratinocyte differentiation was one of the top GO biological functions predicted from our transcriptome profile in EoE patients (Figure 2E). Examination of genes involved in keratinocyte differentiation revealed a distinct enrichment of small proline-rich protein (*SPRR*) genes, as 7 out of 29 genes were significantly downregulated (Figure 3A). ADVAITA iPathwayGuide analysis reveals downstream implications of keratinocyte differentiation on epidermal cell differentiation that further affect epithelium development, cell differentiation, and anatomical structure development (Figure 3B). We next analyzed our whole-transcriptome dataset specifically for *SPRR* genes and found that all 10 *SPRR* genes were downregulated with 7 genes identified as significant (Figure 3C). GO biological analysis of the *SPRR* gene family predicts the involvement of these genes in keratinization, keratinocyte/epidermal/epithelial cell differentiation, skin/epidermis/epithelium development, and peptide cross-linking (Figure 3D). These biological functions are expected to occur within the cornified envelope (Figure 3E). Reactome pathway prediction further reinforces the implications genetic dysregulation has on formation of the cornified envelope, keratinization, and developmental biology (Figure 3F).

### Impaired *MUC* gene expression in EoE biopsies

**Figure 4:**
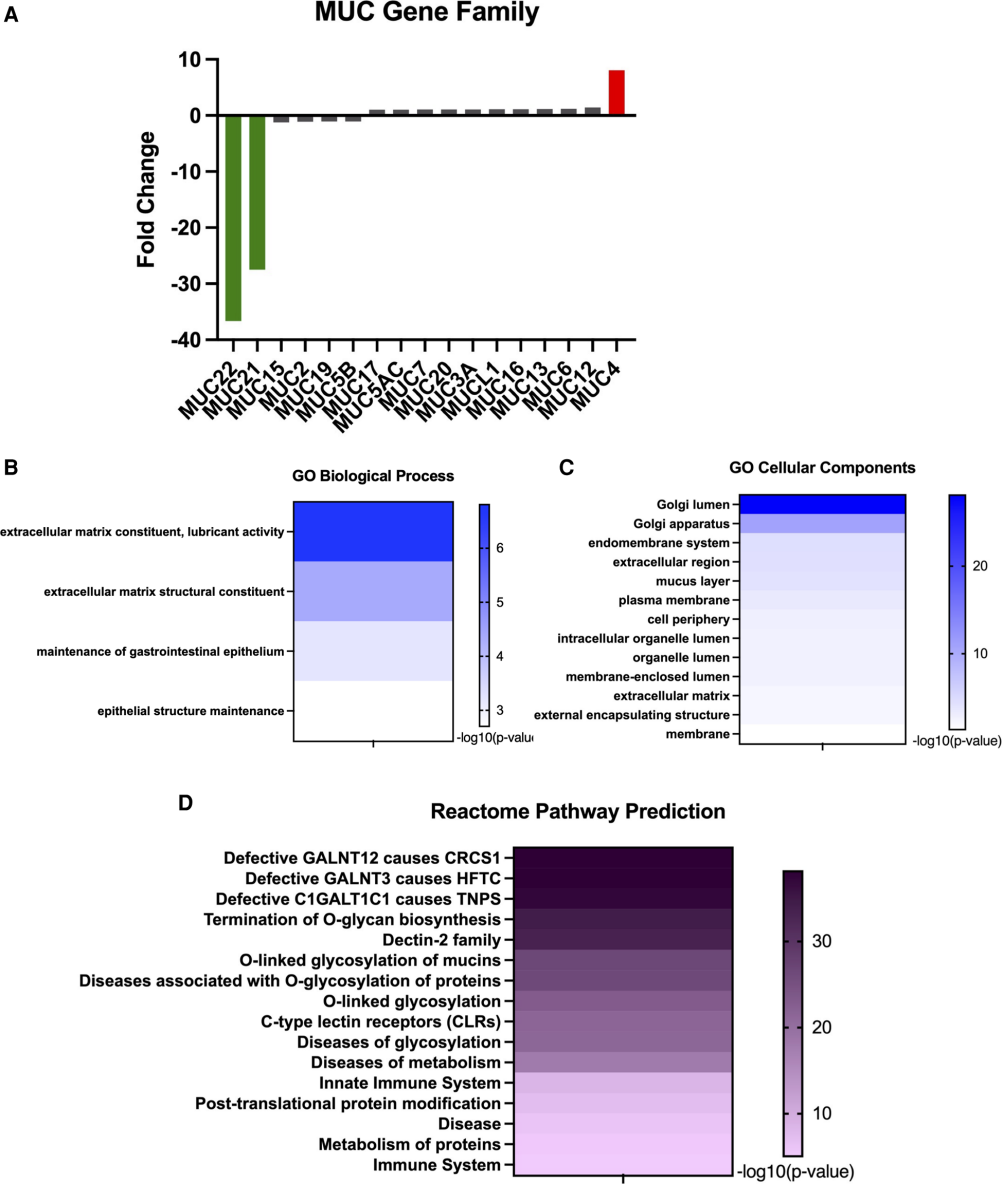
Unique expression of MUC genes in EoE samples. (**A**) Gene expression of MUC gene family. (**B**) GO: biological processes for MUC gene family. (**C**) GO: cellular components for MUC family. (**D**) Reactome pathway prediction for MUC gene Family.

Mucin expression is a common biomarker for several diseases with interference in production impairing cellular integrity. Within the gastrointestinal tract, the amount of mucins generally increases from esophagus to rectum (28). Under normal physiologic states, the human esophagus produces *MUC1, MUC4, MUC5B*, and *MUC20* (28). Evaluation of the *MUC* genes indicated that two genes were significantly downregulated: *MUC22* (−36.63-fold) and *MUC21* (−27.49-fold) (Figure 4A). *MUC4* was the only gene in this family found to be significantly upregulated (8.08-fold) (Figure 4A). To date, these mucins have not been evaluated in the context of eosinophilic esophagitis. However, MUC4 is associated with normal squamous epithelium; therefore, an increase in this gene expression may be indicative of increased differentiation. However, increased MUC4 has also been observed other diseased esophagus states, including Barrett's esophagus and high-grade intraepithelial neoplasia (29). A glycoform of MUC21 with extended carbohydrate chains was found in suprabasal cells and serves as a marker for differentiation in squamous cell carcinoma (30), which suggests another possible marker for esophageal differentiation. Dysregulation of these mucin genes are expected to impact extracellular matrix constituents, particularly involving lubricant activity and structure (Figure 4B). GO cellular components largely predicts this dysregulated activity to occur within the Golgi lumen (Figure 4C), the site at which mucin dimers move from the ER for assembling of *O*-glycans. As the type and composition of mucins are indicative of tissue, physiologic state, and pathology, mucins contain a spectacle of *O*-linked oligosaccharides that vary between the types of mucins, tissues, and pathologic conditions and is predicted to vary between EoE patients and Controls (Figure 4D).

### Overall loss of epidermal differentiation genes in EoE biopsies

**Figure 5:**
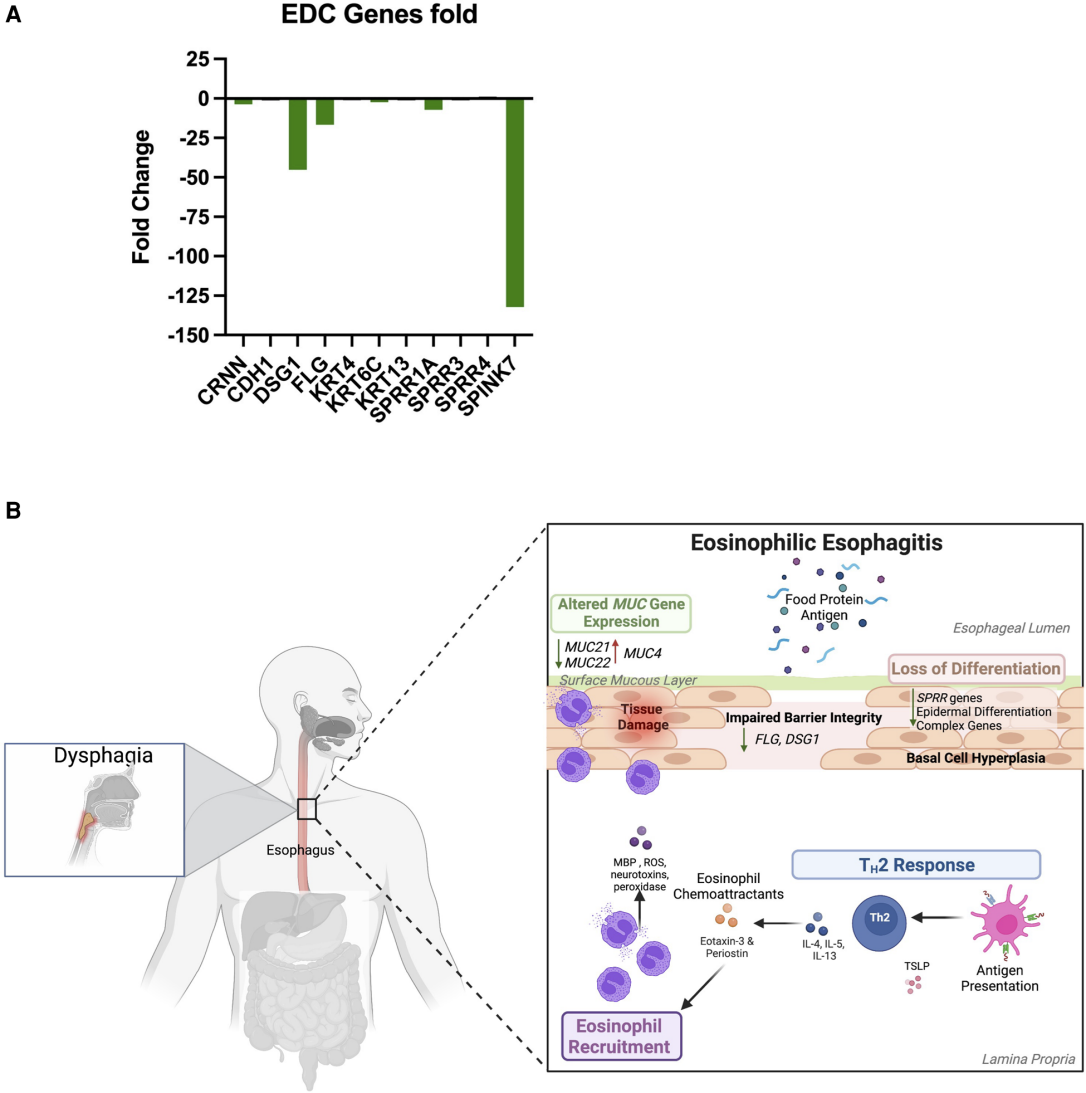
Proposed model for EoE. (**A**) Gene expression related to epidermal differentiation complex (EDC). (**B**) EoE biopsies are characterized by dysregulation of SPRR genes and MUC21, which are predicted to impair differentiation of squamous epithelial cells and further impair their capacity to cope with prolonged inflammatory conditions.

Previous work from the Human Protein Atlas evaluated esophagus-specific transcripts and found that transcripts were predicted to implicate keratinization and differentiation. This unique transcriptome profile was correlated with EoE biopsies lacking adequate tissue differentiation (27). Our work also found common genes related to keratinization and differentiation to be DEGs. Cornulin expression was found to be downregulated in our dataset comparing EoE biopsies to Controls (*CRNN*, −3.69-fold) (Figure 5A). Filaggrin *(FLG*, −16.63-fold) and desmoglein 1 (*DSG-1,* −132.09-fold) were also down-regulated in EoE patients (structural integrity of the epithelial barrier) (Figure 5A). Further, a member of the SPRR family, *SPINK7* was decreased (−132.09-fold). Keratin-6C (*KRT6C)* was found to be downregulated at −2.35 (Figure 5A). This is consistent with our transcriptome profile for EoE patients as functional enrichment GO predicted impaired biological functions related to keratinization, differentiation, and epidermis/epithelium/tissue development (Figure 3D). Reactome analysis further predicted formation of the cornified envelope to be impacted (Figure 3F). Transcriptome analysis of the EDC genes reveals an overall downregulation of genes related to cornification, proper maturation, and terminal differentiation of squamous epithelial cells.

## Discussion

The dataset we present supports previously reported transcriptomic profiles associated with EoE disease states while also offering novel insight by identifying genes previously unreported with EoE. Periostin (*POSTN*) was significantly upregulated and desmoglein 1 (*DSG1*) downregulated compared to Controls in our cohort. These findings support previous models of EoE pathogenesis that describe the interplay of these two genes creating a paradigm induced by IL-13 in which decreased desmoglein function disrupts the epithelial barrier, permitting increased eosinophil adhesion mediated by overactive periostin (14, 15). Another gene, *TSLP*, has also been tied to this inflammatory mechanism by promoting T_H_2 differentiation and the pro-inflammatory cycle (31, 32), though this gene was not significantly downregulated in our dataset (fold change −1.07). While this finding is inconsistent with previous reports, several environmental factors have been noted to influence the pathogenesis of EoE. In adults, smoking and other aeroallergens drive T_H_2 inflammation (33), whereas in children early antibiotic treatment, cesarean delivery, and preterm birth were noted as important for pathogenesis (34, 35). The gene perhaps most commonly tied to EoE, eotaxin-3 (*CCL26*), was present among our greatly upregulated genes. Studies have described this gene locus as being under significant epigenetic influence (13, 36). Further investigation of other gene loci may reveal the importance of epigenetics among other markers of EoE. The wide age distribution of our cohort may play a role in the expression levels of classic EoE genes in our analysis given the variable effects of epigenetics and environmental exposures at different ages. It is important to note that Control biopsies collected for this study were collected from patients undergoing exploratory endoscopy for another disease/condition independent of EoE. As a result, a limitation of this study is that Controls were collected from patients that presented with symptoms analogous to EoE but were confirmed non-EoE given histopathological assessment.

Seminal work conducted in 2017 evaluated genes from the Human Protein Atlas to compare esophagus-specific transcripts as they may relate to EoE pathogenesis (27). They found approximately 39% of esophagus-specific transcripts were dysregulated in EoE patients (27) These transcripts were predicted to implicate keratinization/differentiation and correlated with EoE biopsies lacking adequate tissue differentiation (27). This is consistent with our transcriptome profile for EoE patients as functional enrichment GO predicted impaired biological functions related to keratinization, differentiation, and epidermis/epithelium/tissue development (Figure 3D). Reactome analysis further predicted formation of the cornified envelope to be impacted (Figure 3F). Although there are overlaps in functions between the skin and mucosal surfaces of the gut in forming an effective immune barrier from the external environment, these tissues have distinct anatomical differences. For instance, terminal differentiation of epidermal keratinocytes in the skin results in desquamation and cornified cells at the surface. It is important to note that cornification is atypical within the normal esophageal epithelium, where it is instead composed of nonkeratinized stratified squamous epithelium (37). Beyond the evaluation of the SPRR gene family in EDC, little more is known about its involvement in the normal esophagus or EoE. Here, we document several differentially expressed SPRR genes in human EoE (Figure 3C). Work conducted in 2021 analyzed the interplay of SPRR genes and late cornified envelope (LCE) genes and found co-localization with SPRR2 and a member of the LCE3D in psoriasis (38). *SPRR2A, SPRR2B, SPRR2D, SPRR2E,* and *SPRR2F* were all found to be decreased in EoE biopsies (Figure 3C)*.* An emerging school of thought suggests a gut-skin axis linking skin and gut disorders, with the common denominator being dysregulated microbiomes driven by environmental factors, suggesting that more visually apparent skin disorders may give insight to systemic issues occurring simultaneously within the gut (39). We previously showed in a spontaneous murine model for hypereosinophilic-like syndrome that mice lacking noncanonical NF-*κ*B signaling had systemic eosinophilia, including atopic dermatitis and esophagitis (40). Concomitant atopic dermatitis has also been reported in 2–19% human EoE patient (41). We suggest that a better understanding of EoE pathogenesis may be derived from reinterpretations of the well-studied involvement of the *SPRR* gene family and its implications on cornification.

Current work has sought to better evaluate proper differentiation of epithelial cells within the basal zone (undifferentiated, proliferating cells) and suprabasal zone (differentiated cells) of the esophagus (37, 42). Transcriptome analysis of EDC genes reveals suprabasal zone cells have increased expression of the keratins *KRT4* and *KRT13* (27, 43–45). This work sought to evaluate specific epithelial differentiation markers given pathophysiologic conditions (27). They found increased undifferentiated markers (*KRT5*, *KRT14)* and decreased terminal differentiation marker *(KRT4*) in active EoE patients (27). Although highly expressed in the normal esophagus, decreased cornulin (*CRNN*) expression is observed in EoE patients (27). This was consistent with our dataset comparing (*CRNN*, −3.69-fold) (Figure 5A). Other groups further assessed EDC genes as they relate to drug responsiveness (37) and found filaggrin to be down-regulated 16-fold in EoE patients, which is identical to our results (*FLG*, −16.63-fold) (Figure 5A). Following treatment with glucocorticoids and/or dietary changes, fillagrin expression was not significantly changed, indicating EDC genes can be reversed following responsiveness to treatment (37). This suggests improper differentiation within the esophagus originates at a genetic level and may cause EoE patients to be susceptible to irregular anatomical development.

Previous work recorded distinct changes in mucin gene expression and production. Within the gastrointestinal tract, the amount of mucins generally increases from esophagus to rectum (28). Mucins produced by the esophagus under normal physiologic states include MUC1, MUC4, MUC5B, and MUC20 (28). MUC1 and MUC4 are expressed in normal squamous epithelium, while MUC5B is expressed by submucosal glands (46). Discrepancy in mucin production can also be associated with pathologic features of the esophagus. For instance, MUC1 and MUC4 levels correlate to the degree of proper differentiation occurring within the epithelia (46), whereas MUC5AC has been associated with tissue remodeling in the lung and found to be upregulated in human EoE patients (47, 48). In our dataset, we found *MUC4* to be increased 8.08-fold, while *MUC1* and *MUC5AC* were not differentially expressed (Figure 4A). Another group found *MUC1* and *MUC5B* expression to be downregulated, while *MUC4* was increased (49). We did not find *MUC5B* to be significantly varied in our dataset (Figure 4A). Two MUC genes, *MUC21* (−36.63-fold) and *MUC22* (−27.49-fold), were uniquely altered in our transcriptome profiles (Figure 4A)*.* To date, MUC22 has not been evaluated in the context of the esophageal epithelium. However, a glycoform of MUC21 with extended carbohydrate chains was found in suprabasal cells and serves as a marker for differentiation in squamous cell carcinoma (30), indicating a potential role of MUC21 *O*-glycosylation in esophageal differentiation, which would be consistent with Reactome pathway predictions (Figure 4D). Altered mucin production further recapitulates that improper differentiation occurring within the epithelium may have deleterious downstream effects on barrier function, tissue remodeling, and overall cell integrity within this tissue to culminate in disease and sensitizing this tissue to food allergens.

Our data contributes additional insight into the transcriptome profile of EoE patients and contributes to our understanding of EoE pathogenesis. We highlight two families of genes (*SPRR*, *MUC*) that are understudied in the context of EoE and suggest their involvement in improper epithelial cell differentiation of the diseased esophagus. Information culminated from this study suggests potential targets for therapeutic development focusing on EDC. Current treatment options resolve pro-inflammatory signaling to remediate symptoms without directly addressing specific dysfunctional biological processes, thus necessitating an urgency to develop improved options for EoE. Our data suggest that SPRR and/or MUC are potential targets to resolve EDC and markers of improper tissue damage responses that may be viable therapeutic options. Furthermore, we propose the *SPRR* and *MUC* gene families, as well as several other previously identified genes also found in our analysis, are a promising set of genes potentially associated with disease etiology (*CCL26, CPA3, POSTN, CTSC, ANO1, CRISP3, SPINK7).* With more mechanistic insight, these genes may become useful for clinical evaluation of EoE as part of a genetic panel. Culminating our research findings, we highlight both the clinical and translational relevancy of the unique EoE gene expression profile with the hope of one day improving EoE diagnosis, detection, and treatment.

## Data Availability

The datasets presented in this study can be found in online repositories. The names of the repository/repositories and accession number(s) can be found below: https://www.ncbi.nlm.nih.gov/geo/, GSE228083.

## References

[1] Lucendo AJ Molina-Infante J Arias A von Arnim U Bredenoord AJ Bussmann C Guidelines on eosinophilic esophagitis: evidence-based statements and recommendations for diagnosis and management in children and adults. United European Gastroenterol J. (2017) 5(3):335–58. 10.1177/2050640616689525 28507746 PMC5415218

[2] Ahmed M. Eosinophilic esophagitis in adults: an update. World J Gastrointest Pharmacol Ther. (2016) 7(2):207–13. 10.4292/wjgpt.v7.i2.207 27158535 PMC4848242

[3] Hruz P. Epidemiology of eosinophilic esophagitis. Dig Dis. (2014) 32(1–2):40–7. 10.1159/000357008 24603379

[4] Dellon ES Hirano I. Epidemiology and natural history of eosinophilic esophagitis. Gastroenterology. (2018) 154(2):319–32.e3. 10.1053/j.gastro.2017.06.067 28774845 PMC5794619

[5] Jensen ET Dellon ES. Environmental and infectious factors in eosinophilic esophagitis. Best Pract Res Clin Gastroenterol. (2015) 29(5):721–9. 10.1016/j.bpg.2015.06.008 26552771 PMC4641821

[6] Stuck MC Straumann A Simon HU. Relative lack of T regulatory cells in adult eosinophilic esophagitis—no normalization after corticosteroid therapy. Allergy. (2011) 66(5):705–7. 10.1111/j.1398-9995.2010.02525.x 21470243

[7] Lingblom C Kappi T Bergquist H Bove M Arkel R Saalman R Differences in eosinophil molecular profiles between children and adults with eosinophilic esophagitis. Allergy. (2017) 72(9):1406–14. 10.1111/all.13140 28194801

[8] Straumann A Aceves SS Blanchard C Collins MH Furuta GT Hirano I Pediatric and adult eosinophilic esophagitis: similarities and differences. Allergy. (2012) 67(4):477–90. 10.1111/j.1398-9995.2012.02787.x 22313241

[9] Gonsalves NP Aceves SS. Diagnosis and treatment of eosinophilic esophagitis. J Allergy Clin Immunol. (2020) 145(1):1–7. 10.1016/j.jaci.2019.11.011 31910983 PMC6986782

[10] Greuter T Hirano I Dellon ES. Emerging therapies for eosinophilic esophagitis. J Allergy Clin Immunol. (2020) 145(1):38–45. 10.1016/j.jaci.2019.10.027 31705907 PMC6981295

[11] Dellon ES Rothenberg ME Collins MH Hirano I Chehade M Bredenoord AJ Dupilumab in adults and adolescents with eosinophilic esophagitis. N Engl J Med. (2022) 387(25):2317–30. 10.1056/NEJMoa2205982 36546624

[12] Roufosse F Kahn JE Rothenberg ME Wardlaw AJ Klion AD Kirby SY Efficacy and safety of mepolizumab in hypereosinophilic syndrome: a phase iii, randomized, placebo-controlled trial. J Allergy Clin Immunol. (2020) 146(6):1397–405. 10.1016/j.jaci.2020.08.037 32956756 PMC9579892

[13] Blanchard C Wang N Stringer KF Mishra A Fulkerson PC Abonia JP Eotaxin-3 and a uniquely conserved gene-expression profile in eosinophilic esophagitis. J Clin Invest. (2006) 116(2):536–47. 10.1172/JCI26679 16453027 PMC1359059

[14] Sherrill JD Kc K Wu D Djukic Z Caldwell JM Stucke EM Desmoglein-1 regulates esophageal epithelial barrier function and immune responses in eosinophilic esophagitis. Mucosal Immunol. (2014) 7(3):718–29. 10.1038/mi.2013.90 24220297 PMC3999291

[15] Sherrill JD Rothenberg ME. Genetic and epigenetic underpinnings of eosinophilic esophagitis. Gastroenterol Clin North Am. (2014) 43(2):269–80. 10.1016/j.gtc.2014.02.003 24813515 PMC4019933

[16] Azouz NP Ynga-Durand MA Caldwell JM Jain A Rochman M Fischesser DM The antiprotease Spink7 serves as an inhibitory checkpoint for esophageal epithelial inflammatory responses. Sci Transl Med. (2018) 10(444). 10.1126/scitranslmed.aap9736 29875205 PMC6065103

[17] Cianferoni A Ruffner MA Guzek R Guan S Brown-Whitehorn T Muir A Elevated expression of activated T(H)2 cells and milk-specific T(H)2 cells in milk-induced eosinophilic esophagitis. Ann Allergy Asthma Immunol. (2018) 120(2):177–83.e2. 10.1016/j.anai.2017.11.006 29289462 PMC5875940

[18] Rochman M Xie YM Mack L Caldwell JM Klingler AM Osswald GA Broad transcriptional response of the human esophageal epithelium to proton pump inhibitors. J Allergy Clin Immunol. (2021) 147(5):1924–35. 10.1016/j.jaci.2020.09.039 33289661 PMC8062577

[19] Hirano I Moy N Heckman MG Thomas CS Gonsalves N Achem SR. Endoscopic assessment of the oesophageal features of eosinophilic oesophagitis: validation of a novel classification and grading system. Gut. (2013) 62(4):489–95. 10.1136/gutjnl-2011-301817 22619364

[20] Gonsalves N Berdnikovs S Schroeder H Zalewski A Bryce PJ. Gender-specific differences in the molecular signatures of adult eosinophilic oesophagitis. Clin Exp Allergy. (2017) 47(7):969–71. 10.1111/cea.12960 28580626 PMC5862544

[21] Blanchard C Stucke EM Rodriguez-Jimenez B Burwinkel K Collins MH Ahrens A A striking local esophageal cytokine expression profile in eosinophilic esophagitis. J Allergy Clin Immunol. (2011) 127(1):208–17; 17.e1–7. 10.1016/j.jaci.2010.10.039 21211656 PMC3027004

[22] Kottyan LC Rothenberg ME. Genetics of eosinophilic esophagitis. Mucosal Immunol. (2017) 10(3):580–8. 10.1038/mi.2017.4 28224995 PMC5600523

[23] Vanoni S Zeng C Marella S Uddin J Wu D Arora K Identification of anoctamin 1 (Ano1) as a key driver of esophageal epithelial proliferation in eosinophilic esophagitis. J Allergy Clin Immunol. (2020) 145(1):239–54.e2. 10.1016/j.jaci.2019.07.049 31647967 PMC7366251

[24] Niranjan R Rajavelu P Ventateshaiah SU Shukla JS Zaidi A Mariswamy SJ Involvement of interleukin-18 in the pathogenesis of human eosinophilic esophagitis. Clin Immunol. (2015) 157(2):103–13. 10.1016/j.clim.2015.01.007 25638412 PMC4580377

[25] Whelan KA Merves JF Giroux V Tanaka K Guo A Chandramouleeswaran PM Autophagy mediates epithelial cytoprotection in eosinophilic oesophagitis. Gut. (2017) 66(7):1197–207. 10.1136/gutjnl-2015-310341 26884425 PMC4987278

[26] Doshi A Khamishon R Rawson R Duong L Dohil L Myers SJ Interleukin 9 alters epithelial barrier and E-cadherin in eosinophilic esophagitis. J Pediatr Gastroenterol Nutr. (2019) 68(2):225–31. 10.1097/MPG.0000000000002144 30211842 PMC6344288

[27] Rochman M Travers J Miracle CE Bedard MC Wen T Azouz NP Profound loss of esophageal tissue differentiation in patients with eosinophilic esophagitis. J Allergy Clin Immunol. (2017) 140(3):738–49.e3. 10.1016/j.jaci.2016.11.042 28104354 PMC5513800

[28] Andrianifahanana M Moniaux N Batra SK. Regulation of mucin expression: mechanistic aspects and implications for cancer and inflammatory diseases. Biochim Biophys Acta. (2006) 1765(2):189–222. 10.1016/j.bbcan.2006.01.002 16487661

[29] Bax DA Haringsma J Einerhand AW van Dekken H Blok P Siersema PD Muc4 is increased in high grade intraepithelial neoplasia in Barrett's oesophagus and is associated with a proapoptotic bax to Bcl-2 ratio. J Clin Pathol. (2004) 57(12):1267–72. 10.1136/jcp.2004.017020 15563666 PMC1770513

[30] Tian Y Denda-Nagai K Kamata-Sakurai M Nakamori S Tsukui T Itoh Y Mucin 21 in esophageal squamous epithelia and carcinomas: analysis with glycoform-specific monoclonal antibodies. Glycobiology. (2012) 22(9):1218–26. 10.1093/glycob/cws082 22611128

[31] Kitajima M Lee HC Nakayama T Ziegler SF. Tslp enhances the function of helper type 2 cells. Eur J Immunol. (2011) 41(7):1862–71. 10.1002/eji.201041195 21484783 PMC3124605

[32] O'Shea KM Aceves SS Dellon ES Gupta SK Spergel JM Furuta GT Pathophysiology of eosinophilic esophagitis. Gastroenterology. (2018) 154(2):333–45. 10.1053/j.gastro.2017.06.065 28757265 PMC5787048

[33] Slae M Persad R Leung AJ-T Gabr R Brocks D Huynh HQ. Role of environmental factors in the development of pediatric eosinophilic esophagitis. Dig Dis Sci. (2015) 60(11):3364–72. 10.1007/s10620-015-3740-7 26062820

[34] Lyles J Rothenberg M. Role of genetics, environment, and their interactions in the pathogenesis of eosinophilic esophagitis. Curr Opin Immunol. (2019) 60:46–53. 10.1016/j.coi.2019.04.004 31132551 PMC6800613

[35] Jensen ET Kuhl JT Martin LJ Langefeld CD Dellon ES Rothenberg ME. Early-life environmental exposures interact with genetic susceptibility variants in pediatric patients with eosinophilic esophagitis. J Allergy Clin Immunol. (2018) 141(2):632–7.e5. 10.1016/j.jaci.2017.07.010 29029802 PMC5803324

[36] Lim E Rothenberg ME. Demethylation of the human eotaxin-3 gene promoter leads to the elevated expression of eotaxin-3. J Immunol. (2014) 192(1):466–74. 10.4049/jimmunol.1302454 24323578 PMC3902544

[37] Blanchard C Stucke EM Burwinkel K Caldwell JM Collins MH Ahrens A Coordinate interaction between Il-13 and epithelial differentiation cluster genes in eosinophilic esophagitis. J Immunol. (2010) 184(7):4033–41. 10.4049/jimmunol.0903069 20208004 PMC3807813

[38] Tian S Chen S Feng Y Li Y. The interactions of small proline-rich proteins with late cornified envelope proteins are involved in the pathogenesis of psoriasis. Clin Cosmet Investig Dermatol. (2021) 14:1355–65. 10.2147/CCID.S336072 34594126 PMC8478164

[39] De Pessemier B Grine L Debaere M Maes A Paetzold B Callewaert C. Gut-skin axis: current knowledge of the interrelationship between microbial dysbiosis and skin conditions. Microorganisms. (2021) 9(2). 10.3390/microorganisms9020353 33670115 PMC7916842

[40] Eden K Rothschild DE McDaniel DK Heid B Allen IC. Noncanonical nf-kappab signaling and the essential kinase nik modulate crucial features associated with eosinophilic esophagitis pathogenesis. Dis Model Mech. (2017) 10(12):1517–27. 10.1242/dmm.030767 29259025 PMC5769607

[41] Daley A Lehman E Jhaveri P. Association of initial esophageal eosinophil counts with atopic dermatitis in patients with eosinophilic esophagitis. Ann Allergy Asthma Immunol. (2021) 127(6):696–7. 10.1016/j.anai.2021.09.003 34537359

[42] Rochman M Azouz NP Rothenberg ME. Epithelial origin of eosinophilic esophagitis. J Allergy Clin Immunol. (2018) 142(1):10–23. 10.1016/j.jaci.2018.05.008 29980278 PMC8034427

[43] Jeong Y Rhee H Martin S Klass D Lin Y Nguyen le XT Identification and genetic manipulation of human and mouse oesophageal stem cells. Gut. (2016) 65(7):1077–86. . 10.1136/gutjnl-2014-308491 25897018

[44] Zhang Y Jiang M Kim E Lin S Liu K Lan X Development and stem cells of the esophagus. Semin Cell Dev Biol. (2017) 66:25–35. 10.1016/j.semcdb.2016.12.008 28007661 PMC5474349

[45] Okumura T Shimada Y Imamura M Yasumoto S. Neurotrophin receptor P75(Ntr) characterizes human esophageal keratinocyte stem cells in vitro. Oncogene. (2003) 22(26):4017–26. 10.1038/sj.onc.1206525 12821936

[46] Guillem P Billeret V Buisine MP Flejou JF Lecomte-Houcke M Degand P Mucin gene expression and cell differentiation in human normal, premalignant and malignant esophagus. Int J Cancer. (2000) 88(6):856–61. 10.1002/1097-0215(20001215)88:6<856::aid-ijc3>3.0.co;2-d 11093805

[47] Mishra A Wang M Pemmaraju VR Collins MH Fulkerson PC Abonia JP Esophageal remodeling develops as a consequence of tissue specific Il-5-induced eosinophilia. Gastroenterology. (2008) 134(1):204–14. 10.1053/j.gastro.2007.10.002 18166354 PMC2654267

[48] Sueyoshi S Miyata Y Masumoto Y Ishibashi Y Matsuzawa S Harano N Reduced airway inflammation and remodeling in parallel with mucin 5ac protein expression decreased by S-carboxymethylcysteine, a mucoregulant, in the airways of rats exposed to sulfur dioxide. Int Arch Allergy Immunol. (2004) 134(4):273–80. 10.1159/000079164 15205558

[49] Arias A Vicario M Bernardo D Olalla JM Fortea M Montalban-Arques A Toll-like receptors-mediated pathways activate inflammatory responses in the esophageal mucosa of adult eosinophilic esophagitis. Clin Transl Gastroenterol. (2018) 9(4):147. 10.1038/s41424-018-0017-4 29691386 PMC5915448

