# Supplementary material for: Expression profiling identifies key genes and biological functions associated with eosinophilic esophagitis in human patients
Source: Front Allergy. 2023 Aug 24;4:1239273. doi: 10.3389/falgy.2023.1239273 (PMC10484407; doi:10.3389/falgy.2023.1239273)
Supplement: Supplementary file 1 [file Presentation1.pptx]

## Slide 1
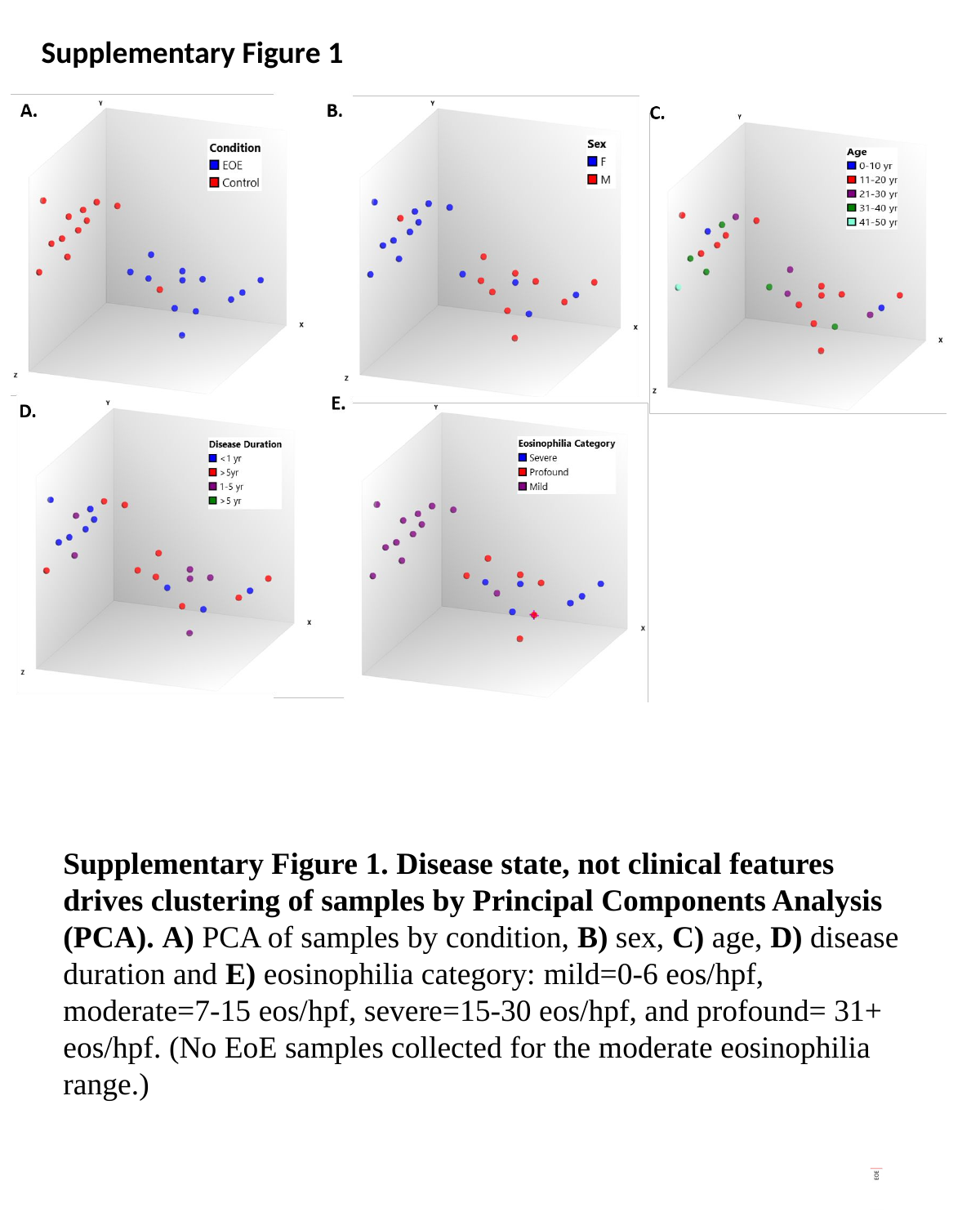

Supplementary Figure 1
Supplementary Figure 1. Disease state, not clinical features drives clustering of samples by Principal Components Analysis (PCA). A) PCA of samples by condition, B) sex, C) age, D) disease duration and E) eosinophilia category: mild=0-6 eos/hpf, moderate=7-15 eos/hpf, severe=15-30 eos/hpf, and profound= 31+ eos/hpf. (No EoE samples collected for the moderate eosinophilia range.)

## Slide 2
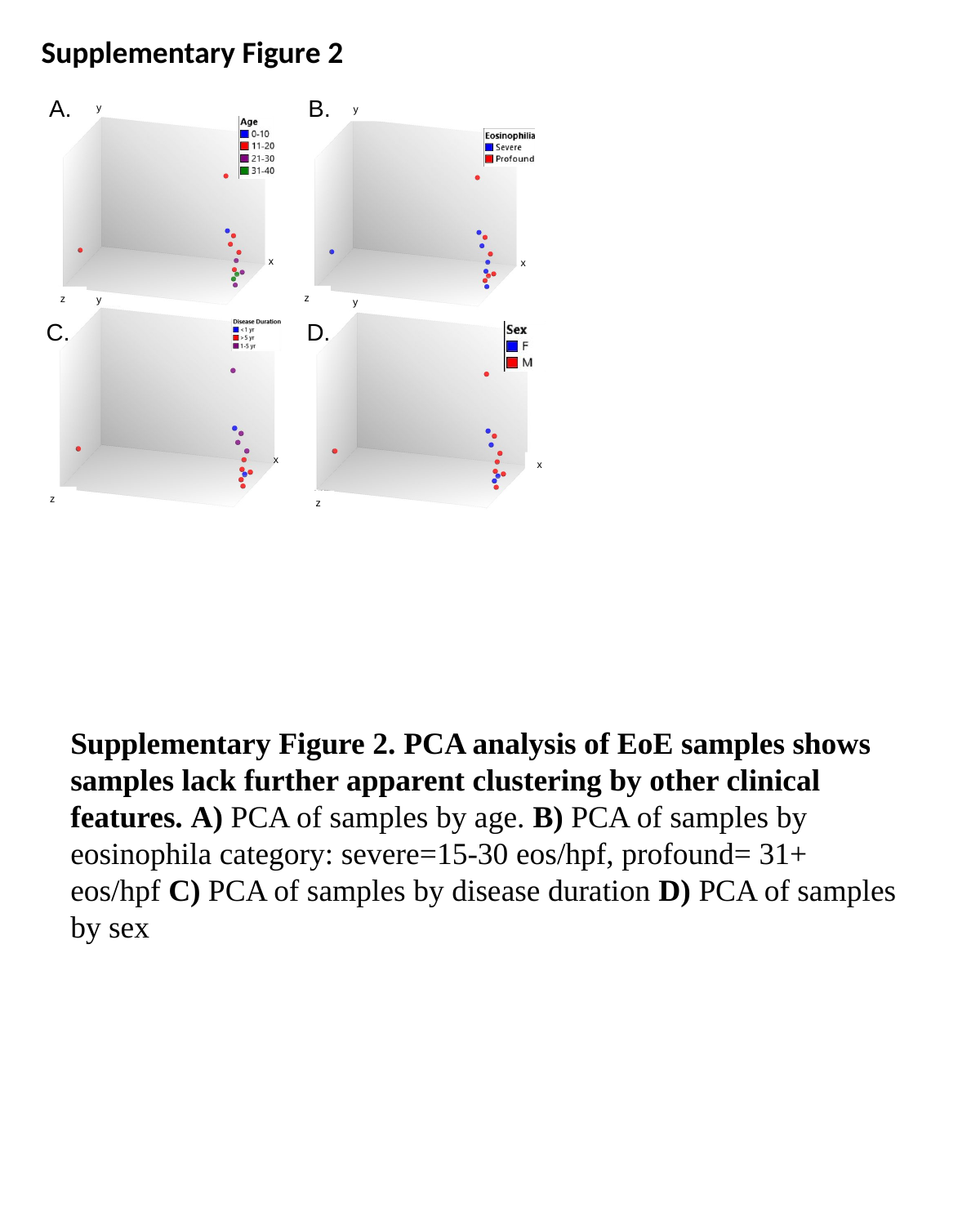

Supplementary Figure 2
B.
A.
y
y
x
x
z
z
y
y
x
z
C.
D.
x
z
Supplementary Figure 2. PCA analysis of EoE samples shows samples lack further apparent clustering by other clinical features. A) PCA of samples by age. B) PCA of samples by eosinophila category: severe=15-30 eos/hpf, profound= 31+ eos/hpf C) PCA of samples by disease duration D) PCA of samples by sex
